# Strategies to Promote Broad-Based Implementation of Acute Care for Elders (ACE) Units

**DOI:** 10.3390/geriatrics3030058

**Published:** 2018-09-06

**Authors:** Roger Y. Wong

**Affiliations:** Division of Geriatric Medicine, Department of Medicine, Faculty of Medicine, University of British Columbia, Vancouver, BC V5Z 1M9, Canada; roger.wong@ubc.ca

It is widely accepted that older people are at increased risk of functional decline when they present to acute care hospitals [1], and as a result, a number of best practice models of care have been proposed to mitigate the risk [2]. One of these models is the Acute Care for Elders (ACE) unit [3]. In this issue of the Journal, Palmer presents a comprehensive and contemporary review of the topic, which describes the rationale, development, and scientific evidence of ACE units [4]. A thorough read of the review is very worthwhile.

Despite the evidence, the implementation of ACE units has not been broad-based, at least from a global perspective. In contrast, widespread implementation of other specialized units has long become the established norm in acute care hospitals, such as intensive care units for critically ill patients, coronary care units for patients with acute coronary syndromes, and stroke units for patients with cerebrovascular accidents, to name a few. In these examples, the specialized units represent the quality benchmarks in serving special patient care needs. If we apply the same principle to older people who present with geriatric syndromes compounded by complex acute conditions, we should expect to witness the broad-based implementation of ACE units. This is, however, far from the truth, and the underlying barriers warrant careful and thoughtful reflection.

A common barrier identified is how the potential benefits of ACE units get communicated to key stakeholders, namely, the policy-makers and administrators within hospitals and health authorities [5]. In a way, this is a form of effective knowledge translation. I would like to take a moment to articulate five key messages that can help to promote the broad-based implementation of the ACE model of care.

1. ACE can improve patient outcomes.

In the era of evidence-based practice, it is important to highlight the positive effects of the ACE model of care on improving patient outcomes, namely in reducing functional decline, reducing iatrogenic adverse events (such as falls, skin breakdown, and delirium), and promoting home discharges to the community [6].

2. ACE is efficient care.

The ACE model of care has been around since the 1990s, and they can add to efficiency of care by reducing the mean hospital length of stay. In a recent meta-analysis, the mean reduction is 1.28 days, which is statistically significant [6]. 

3. ACE can be cost saving (at least cost neutral).

The economic benefit of ACE has raised questions among critics, namely due to the absence of a definitive, large-scale cost-effectiveness study thus far. The shorter length of hospital stay associated with ACE described above can translate to savings [6], although there can be initial costs in renovating older hospital units and retraining staff [4]. The overall economic impact of ACE is more likely to be cost neutral.

4. ACE can identify improvement opportunity in patient care.

There are numerous clinical quality improvement opportunities made possible in the ACE model of care, such as medication reconciliation, minimal/no physical restraint use, and bowel and bladder continence care, to name a few. To achieve this, try to establish a formal connection with the hospital data analytics service so that improvement initiatives can become part of routine practice.

5. The success of ACE requires adequate workforce capacity.

A prerequisite for a successful ACE model of care is an adequate complement of inter-professional staff, which commonly includes the following disciplines: physicians (geriatrician, internist, hospitalist), nurses and care aides, advanced practice nurses, patient care coordinators, physical therapists, occupational therapists, rehabilitation assistants, social workers, clinical dieticians, pharmacists, spiritual care workers, and transitional service workers (to liaise with community services). The exact number of health professional staff in each discipline depends on the size and scope of ACE, as well as the resourcing available.

In addition to the above key messages, it is also important to bear in mind the five helpful strategies below that would facilitate the impactful delivery of these messages.

1. Be prepared, and have the “elevator pitch” ready at any time.

This strategy may appear intuitive, but is actually easier said than done. Know your messages well, and be ready to deliver it in any place and at any time. The “elevator pitch” refers to the compilation of key messages that can be delivered within a couple of minutes. The occasion to deliver the messaging can be varied, such as during care team meetings, hospital or health authority board meetings, or even hallway conversations.

2. Keep your messaging consistent, and communicate often.

A common pitfall in communicating the benefits of any geriatrics model of care is inconsistent messaging, the scenario of “trying to invent new and more appealing reasons” all the time. The key messages of the ACE model are clear and convincing, so stick with them and try to convey them repeatedly on multiple occasions. 

3. Adopt a scholarly approach to strengthen your messaging.

A scholarly approach (via research or quality improvement studies) to document the benefits of the ACE model can help to strategically strengthen and disseminate the key messages. This is also true for projects conducted by students and trainees. The ACE model can provide an in vivo environment for a number of health service delivery projects. 

4. Be part of the solution, not the problem.

The ACE model of care can be part of the solution for the big-picture healthcare eco-system. In other words, position ACE strategically to be one of the mainstream or “must-have” services provided at the hospital, rather than a boutique or “nice-to-have” add on. A confrontational approach that frames ACE as the problem is not a sound strategy.

5. Align with best practices, whether they are local, national, or international. 

A thorough understanding of the latest developments and best practices in the acute care models of care can create the strategic opportunity to start the dialogue of implementing the ACE model. A recent example is the recently published online tool on how best to organize hospital services for frail older people [7]. In this example from the United Kingdom, the ACE model is an immediate solution that is perfectly aligned with the online tool.

This article highlights five key messages and five accompanying strategic considerations that can help to promote the broad-based implementation of the ACE model of care. The time to act is now.
